# From harm to healing: transforming the lives of victims of conflict-related sexual violence through reparation

**DOI:** 10.1136/bmjgh-2024-017220

**Published:** 2026-03-23

**Authors:** Sunneva Gilmore, Clara Sandoval-Villalba

**Affiliations:** 1 Law/Medicine, Queen’s University Belfast, Belfast, UK; 2 University of Essex, Colchester, UK

**Keywords:** Accountability, Global Health, Child health, Maternal health, Gender-Based Violence

## Abstract

Conflict-related sexual violence (CRSV) generates devastating harms that affect the individual victim (female, male, Lesbian, Gay, Bisexual, Transgender, Intersex, Queer and inclusive of other orientations (LGBTIQ+), disabled, young and elderly), families, communities and the whole fabric of society. CRSV is a public health concern as it can lead to health consequences including physical, psychosocial and stigma-related harms that are exacerbated by a lack of healthcare infrastructure. There has been some progress on understanding the harm, but data gaps prevail due to practical reasons, definitional problems and stigma.

When violations of human rights or humanitarian law take place, diverse harms can occur, and victims have a right to reparation as enshrined in international law. Reparation aims to address, as far as possible, the multiple harms victims suffer and to positively transform their lives. The right to reparation is fulfilled through a victim-centred process and delivered via appropriate forms of reparation: restitution, compensation, rehabilitation, satisfaction measures and guarantees of non-repetition. When implemented in a timely, participatory and inclusive manner, reparations can have a transformative impact on victims of CRSV. The process should be prompt and combined with individual monetary and rehabilitation measures alongside clear institutional and societal reforms to ensure the non-repetition of such violations. Domestic reparation programmes, fully supported by government and other stakeholders such as health professionals, offer the most sustainable framework for achieving these goals.

**Summary box**

- Conflict-related sexual violence (CRSV) is a significant public health concern that can result in physical, psychological and socioeconomic harms.
- Under international law, victims of CRSV have a right to reparation, which should strive to meaningfully improve and transform their lives.
- Rehabilitation, non-repetition and victim-centred participatory processes can trigger transformative reparation.
- The healthcare community has a major role to play in enabling rehabilitation as a form of reparation for victims.

## Introduction

 The United Nations (UN) defines conflict-related sexual violence (CRSV) as, ‘rape, sexual slavery, forced prostitution, forced pregnancy, forced abortion, enforced sterilisation, forced marriage and any other form of sexual violence of comparable gravity perpetrated against women, men, girls or boys that is directly or indirectly linked to a conflict’.^1^ CRSV may constitute a serious violation of human rights or international humanitarian law, triggering an obligation on states or responsible actors to provide reparation for the harm caused. The authors of this article incorporate a health lens to comprehend how harms from CRSV are experienced on an individual and collective level, and how reparations may contribute to tackling harms and the underlying structural drivers.^2^ Therefore, this piece builds on the existing body of literature and policy that seeks to address challenges in designing reparations that are meaningful to victims.

CRSV is under-reported, but there is a growing attention to the spectrum of victims, particularly from vulnerable and marginalised groups.^3^ Many victims prefer to be referred to as survivors; however, the paper will use the term victim to refer also to survivors unless otherwise stated in the sources or evidence cited. To understand the harm, it is important to recognise that men and boys,^4^ LGBTIQ+ individuals, disabled individuals, combatants^5^ and detainees/prisoners of war, as well as ethnic and religious minorities, can experience CRSV. However, CRSV disproportionately affects women and girls due to unequal power dynamics that are often exacerbated during conflict. The UN indicates that in 2023, of 3622 UN-verified and reported cases of CRSV, 3456 (95%) were against women and girls, 145 against men and boys and 21 were against LGBTIQ+ individuals.^6^ These cases do not represent the scale of CRSV due to barriers in reporting, including stigma as well as conflict-related factors.^7^

An inclusive approach to CRSV aims^8^ to comprehensively understand the multifaceted dynamics involved,^9^ revealing a complex interplay of imbalances in power, gender inequality, cultural and social norms and a lack of accountability through societal breakdown of institutions. CRSV is a public health concern due to the potential for negative physical, psychological, socioeconomic and intergenerational impacts. However, due to a gross underestimation of the problem, the extent of health harms is not adequately captured in the literature. Also, CRSV is usually accompanied by other acts of violence that should also be incorporated into reparation efforts.

This article examines the complex harms endured by victims of CRSV, with particular attention to health-related impacts and the challenges of documenting and mapping such harm. It then reflects on how the right to reparation may contribute to positively transforming victims’ lives.

## CHALLENGES TO UNDERSTANDING THE HARM

There are challenges to collecting epidemiological data on CRSV and health outcomes. The impact of CRSV is shaped by a study’s population selection, including country choice and concerns around the feasibility of conducting research in conflict-affected countries or displaced populations. The outcomes made visible will depend on what is being measured, definitions of who qualifies as a victim of CRSV (eg, limited to adult females), and access difficulties given the destruction of health infrastructure. Two systematic reviews on the negative consequences of CRSV included only civilian victims, missing opportunities to understand harms to victims of CRSV within armed groups.^10^ There has been some progress in data collection, but there remains a paucity of systematic data on victims as well as the range of perpetrators (eg, state forces, armed groups, civilians, peacekeepers).^11^ Greater disaggregated data in health research can help better appreciate harms to certain groups. Therefore, results must be interpreted with consideration of selection biases, ethical safeguards and the compounding effects of non-sexual conflict trauma.

Victims seeking medical care may choose not to disclose experiences of CRSV. This may be due to fear of stigma as with Rohingya victims in Cox’s Bazar in Bangladesh or Sudanese victims in Chad. The mandatory reporting laws, as in Colombia, can discourage victims from seeking medical care as health professionals have an obligation to report cases of sexual violence to law enforcement, including cases of children, without the consent of the victim.^12 13^ Victims, in particular men, may use alternative language to CRSV, such as torture or detention, that may not be captured in datasets.^14^ Equally, determining the relationship between CRSV and certain health outcomes can be particularly difficult in settings affected by armed conflict, where poverty, insecurity and lack of medical and other services inhibit health-seeking behaviour.^15^ These challenges have implications for the design of public health responses such as humanitarian assistance and reparation.

## THE HARMS CAUSED BY CRSV

The accurate identification of harms resulting from CRSV is essential to shaping appropriate and comprehensive reparation measures. The health consequences include physical, psychological, socioeconomic, intergenerational and stigma-related harms that are exacerbated by limited access to appropriate healthcare in conflict. Insecurity and breakdown in law and order during armed conflict exacerbate harms.^16 17^ The harms of CRSV can depend on the *type of sexual violence* committed; on *individual factors* (such as the health, age, gender and disability); and *external/structural factors* (healthcare, family support, ongoing stressors, poor sanitation and hygiene). Prompt and adequate medical care can be critical to treat and/or prevent further harm. The first 72–120 hours after an experience of sexual violence can be time sensitive for medical interventions, such as postexposure prophylaxis for HIV and emergency contraception.

A systematic review of literature on sexual violence in armed conflict identified negative health outcomes among victims globally.^10^ According to this dataset, the most reported consequences were pregnancy, traumatic genital injuries/tears and sexually transmitted infections (STI), such as HIV.^10^ However, there remains a need to understand downstream and interconnected harms. Other consequences to reproductive health include urinary dysfunction,^17^ infertility from pelvic STIs, abdominal pain which may become chronic,^18^ pregnancy loss and sexual dysfunction.^19^ For example, a woman may experience complications from an unsafe abortion for a pregnancy as a result of rape, where there are restrictive abortion laws or due to healthcare provider’s beliefs. A victim may also experience psychological harm from forced reporting laws and traumatisation from the involvement of police authorities when this is not desired.^18^ Most studies encompass women and girls, with less information available on the impacts on men and boys as well as transgender and non-binary individuals. The available data on men reports more traumatic anogenital injuries, which can lead to urinary and bowel problems.^20^

Victims also suffer psychological harm from CRSV as well as from the stigma they may encounter in the aftermath. Victims may experience fear, shame, guilt, anger and suicidal ideation.^20^ According to Ba and Bhopal’s systematic review on the consequences of war-related sexual violence, victims experience post-traumatic stress disorder, depression, anxiety, substance abuse and/or other psychological distress symptoms.^10^ As with physical harms, the delay in addressing mental harm can aggravate the victim’s isolation and psychological well-being as well as the ability to exercise and claim their rights.^21^ Family members may suffer psychological harm from witnessing sexual violence, being coerced into participation or through the lasting disruptions such violence inflicts on family life. Mothers of children born of rape may also suffer self-blame, guilt and shame, as documented among many Yazidi survivors of Islamic State of Iraq and Syria (ISIS) sexual enslavement in both Iraq and Syria.^22^

Victims are often rejected by their families, partners and communities.^23^ Stigma becomes a secondary form of victimisation. Women who survive CRSV may be considered unmarriageable.^24^ When victims disclose experiences of CRSV to their partners, it may result in relationship problems, marital dissolution or accusations of adultery.^23^ Men who have suffered sexual violence may be seen as ‘unable to protect themselves’.^24^ LGBTIQ+ individuals can face intersectional discrimination, where sexual violence magnifies existing disparities linked to sexual orientation, gender identity and other social factors. Stigma means that many victims will avoid seeking medical and/or psychological care and other services.

Physical and psychological health issues can limit victims’ ability to participate in family, social, economic and political life and to engage in justice processes. These harms may, in turn, have an impact on access to health services and to underlying determinants of health such as food, water, shelter and income.^24^

## RESPONDING TO CRSV HARMS THROUGH REPARATION

Reparations are measures to acknowledge and remedy serious human rights violations.^25^ States have an obligation to provide remedies and reparation to victims. Remedies can be judicial (court ordered), often complex, victimising, expensive and lengthy in nature.^26^ Alternatively, domestic reparation programmes can be set up by states (out of court). They have the advantage of enabling prompt and effective access to a range of measures for larger victim populations.^26^ Few remedies, to provide reparation (judicial or out of court) have been available to victims of CRSV. Many are waiting for their day in court (judicial remedy), and only a few countries such as Colombia, Peru, Iraq or Kosovo have put in place domestic reparation programmes (administrative remedies) that have included victims of CRSV^27^ (box 1).

Box 1 **The domestic reparation programme in Colombia**

Colombia’s domestic reparation programme, established by Law 1448/2011, aims to provide transformative reparation to victims, including victims of conflict-related sexual violence (CRSV). It includes different forms of reparation for victims of CRSV such as a letter of recognition, one-time compensation (equivalent to 30 times the national minimum salary), access to mental and physical healthcare (rehabilitation), satisfaction measures and collective reparation, and priority access to land restitution, among others.

In Colombia, over 9 million victims are registered for reparation, including more than 40 000 victims of CRSV. Despite its ambition, the programme has faced major implementation challenges. Limited resources hinder compensation and delivery of reparation in a victim-centred manner. Poor coordination and unequal access to services across the country, especially healthcare, disproportionately affects the most vulnerable.

Justice is also considered a form of reparation under the law, but impunity remains widespread. Only 2% of sexual violence cases that have been opened have led to convictions^37^.

Nonetheless, repairing the health and interconnected harms from CRSV as well as tackling the structures that made it possible are complex challenges that require a range of responses, of which reparations is an essential component.

## Reparation should address root causes of CRSV and provide rehabilitation

Reparation aims to bring victims back to the situation they were in before the violation happened (corrective justice), a goal that is questioned by advocates of a transformative approach to reparation.^28 29^ For them, reparation cannot aim to bring victims back to the situation they were in before the violation happened, if that was a situation of discrimination that only reinforces the continuum of violence that permanently risks the lives and physical integrity of those subjected to sexual violence.^28^ Lasting change needs more. A ‘thicker’ concept of reparation is therefore essential,^30^ mixing a comprehensive approach that combines a survivor-centred process with different forms of reparation, including rehabilitation and guarantees of non-repetition.

Non-repetition aims to prevent the recurrence of human rights violations.^25^ Addressing the root causes of sexual violence requires engaging with actors who reinforce patriarchal norms, including through the transformation of masculinities and the revision of educational curricula. It also involves creating spaces—through culture and the arts—to challenge inequality and empowering victims, especially those silenced by violence, to participate fully in social life. CRSV victims have mobilised to play a crucial role in defining what individual healing and systemic change should look like, and how reparation may contribute to these aims.^31^

The UN Guidance Note of the Secretary-General^32^ encourages reparations for CRSV beyond compensation (box 2). However, connectivity between forms of reparation such as rehabilitation and non-repetition remains underexplored. Culturally sensitive rehabilitation measures, including timely and adequate access to health and other services (vocational, educational, legal) are essential for victims to be able to act as rights holders. Rehabilitation can empower victims to speak out, disrupt impunity, seeking justice and challenge the structural conditions that enable sexual violence.

Box 2 **Guiding principle 4**

Reparations should strive to be transformative, including in design, implementation and impact (United Nations Guidance Note of the Secretary-General)

- Transformative reparations aim to tackle the root causes of violations
- For conflict-related sexual violence, transformative reparations need to address cultures of violence against women, girls and other victims

This requires going beyond compensation and rehabilitation, to ensure victims are not placed back in a situation, where similar violations will reoccur, requiring greater equality and efforts to address the long-term needs of victims.

The failure to provide prompt reparation exacerbates victims’ harm. The longer it takes to respond, the more difficult it is to mitigate the consequences of sexual violence. This is particularly felt where CRSV has led to chronic conditions such as HIV infection, reproductive consequences (eg, infertility) or psychological sequelae. Therefore, reparation must be adequate, prompt and effective as reflected in the 2005 UN Basic Principles on the Right to Remedy and Reparations (UNGA Res. 60/147).^25^

There are different ways to ensure this happens even during armed conflict. For example, urgent interim reparation can be provided to victims in a timely manner^32^ (box 3). A well-designed interim reparation policy that prioritises acknowledgement, compensation and timely access to holistic medical and psychological care is essential to enabling victims to begin recovering from the most severe violations.

Box 3 **Urgent interim reparation in Ukraine**

Between 2024 and 2025, a pilot project in Ukraine provided urgent interim reparation to over 600 survivors of conflict-related sexual violence (CRSV).^38^ Led by a partnership between the Global Survivors Fund, the Ukrainian government, survivors, civil society and other stakeholders, the pilot played a key role in prompting Parliament to adopt Law 4067/2024, creating a domestic reparation programme for survivors of CRSV.^38^ The interim reparation included a formal letter acknowledging the violence, a compensation payment of €3000 and referral to health services.

## VICTIMS OF SEXUAL VIOLENCE REQUIRE A TRANSFORMATIVE REPARATION PROCESS

Reparations for CRSV remain difficult to design and implement, given a lack of understanding of the harms, prevailing patriarchal structures as well as ongoing stigma and intersecting discriminations.^33^ In under-resourced or crisis-affected settings, reparations for CRSV are particularly difficult to operationalise due to weak institutional infrastructure, limited funding, insecurity and fragmented service delivery systems. Without coordinated support and sustained investment, even well-intentioned initiatives, as in Colombia, may struggle to move beyond symbolic acknowledgement.

A precondition for reparations is the recognition of responsibility by those who committed and/or ordered the crimes.^27^ Reparation talk in the absence of recognition of responsibility is often rejected by victims of CRSV.^27^ In Kosovo, for example, the then President Ms. Jahjaga, recognised state responsibility during the exhibition for survivors ‘thinking of you’. Similarly, Colombia recognised its responsibility by designating 25 May as the National Day for Victims.

Reparation should be victim centred, taking due account of their views, harms and needs, as well as context. Enabling victims to actively participate in reparation processes is crucial to ensure that they have ownership of the process, that the initiatives taken contribute to the transformation of root causes and their living conditions. Victims require safe and trusted spaces and camouflage techniques if they wish to speak out about their experience and advocate for redress. For example, Yazidi survivors, many of whom suffered sexual slavery, preferred to be recognised as victims of captivity to provide them with protection.^22^ Victims will only come forward if they know that it is safe to do so, and that the likely outcome of the process is worth it. Anonymity and confidentiality measures as well as networks of trust, such as victims’ groups, can be essential to enable participation of victims. For instance, the Kosova Rehabilitation Center for Torture Victims was designated by the government as one of the non-governmental organisation able to support survivors with the completion of applications that are then sent to the Commission for the Verification and Recognition of the Status of Victims of Sexual Violence During the Kosovo War.^34^

Healthcare professionals are also invaluable stakeholders in the design and delivery of reparations by not only delineating consequences, but considering what evidence-based interventions can improve health outcomes.^35^ Thus, consideration should be given to a variety of actors who may positively contribute to reparation. Victims can also benefit from the support of other survivors, such as the Global Network of Victims and Survivors to End Wartime Sexual Violence (SEMA Network), to informally navigate the day-to-day lived experience of the consequences of sexual violence. Victim mobilisation has been integral to the realisation of victims’ rights, and by collaborating with others, they can further mobilise to articulate and demand reparations to transform their lives.

## CONCLUSION

There is a growing interest in researching CRSV from a range of disciplines, which serves to enrich understandings on causes and consequences of CRSV, as well as the diversity of victims of sexual violence. In essence, CRSV generates multifaceted harm to victims, their families, communities and to the social fabric. It allows for new forms of victimisation to take place, as those who suffer it are subjected to stigma and ostracism. Inclusivity of these manifestations of violence is essential within reparation frameworks. There is concerted advocacy around victims of CRSV not being marginalised in reparation processes but prioritised.

All victims of CRSV should be incorporated into public health responses. This inclusion must begin with early humanitarian assistance, extend through to urgent interim reparation and continue into long-term reparation programmes. Victims have a right to reparation now; that right can be transformative if reparations are well designed and implemented.

Reparations are not only about the outcomes but also the process. They provide victims with a healing experience that allows them to exercise their rights. Crafting and implementing reparations are the result of work done by different stakeholders, including health professionals.^36^ Medicine can play a significant part in these developments by bringing to bear greater insight into the harm caused by sexual violence and pathways to repair.^35^

## Data Availability

All data relevant to the study are included in the article.
